# Patterns of Sedentary Time and Quality of Life in Women With Fibromyalgia: Cross-Sectional Study From the al-Ándalus Project

**DOI:** 10.2196/14538

**Published:** 2020-03-19

**Authors:** Blanca Gavilán-Carrera, Víctor Segura-Jiménez, Pedro Acosta-Manzano, Milkana Borges-Cosic, Inmaculada C Álvarez-Gallardo, Manuel Delgado-Fernández

**Affiliations:** 1 Department of Physical Education and Sport Faculty of Sport Sciences University of Granada Granada Spain; 2 Sport and Health University Research Institute (iMUDS) Granada Spain; 3 Physical Activity for Health Promotion Research Group (PA-HELP) Granada Spain; 4 Department of Physical Education Faculty of Education Sciences University of Cádiz Puerto Real, Cádiz Spain; 5 Biomedical Research and Innovation Institute of Cádiz (INiBICA) Research Unit Puerta del Mar University Hospital University of Cádiz Cádiz Spain

**Keywords:** GT3X+, accelerometry, sedentary behavior, symptomatology

## Abstract

**Background:**

Sedentary time (ST) has been associated with detrimental health outcomes in fibromyalgia. Previous evidence in the general population has shown that not only is the total amount of ST harmful but the pattern of accumulation of sedentary behaviors is also relevant to health, with prolonged unbroken periods (ie, bouts) being particularly harmful.

**Objective:**

To examine the association of the patterns of ST with health-related quality of life (HRQoL) in women with fibromyalgia and to test whether these associations are independent of moderate-to-vigorous physical activity (MVPA).

**Methods:**

A total of 407 women (mean 51.4 years of age [SD 7.6]) with fibromyalgia participated. ST and MVPA were measured with triaxial accelerometry. The percentage of ST accumulated in bouts and the frequency of sedentary bouts of different lengths (≥10 min, ≥20 min, ≥30 min, and ≥60 min) were obtained. Four groups combining total ST and sedentary bout duration (≥30 min) were created. We assessed HRQoL using the 36-item Short-Form Health Survey (SF-36).

**Results:**

A greater percentage of ST spent in all bout lengths was associated with worsened physical function, bodily pain, vitality, social function, and physical component summary (PCS) (all *P*<.05). In addition, a higher percentage of ST in bouts of 60 minutes or more was related to worsened physical role (*P*=.04). A higher frequency of bouts was negatively associated with physical function, social function, the PCS (≥30 min and ≥60 min), physical role (≥60 min), bodily pain (≥60 min), and vitality (≥20 min, ≥30 min, and ≥60 min) (all *P*<.05). Overall, for different domains of HRQoL, these associations were independent of MVPA for higher bout lengths. Patients with high total ST and high sedentary bout duration had significantly worsened physical function (mean difference 8.73 units, 95% CI 2.31-15.15; independent of MVPA), social function (mean difference 10.51 units, 95% CI 2.59-18.44; not independent of MVPA), and PCS (mean difference 2.71 units, 95% CI 0.36-5.06; not independent of MVPA) than those with low ST and low sedentary bout duration.

**Conclusions:**

Greater ST in prolonged periods of any length and a higher frequency of ST bouts, especially in longer bout durations, are associated with worsened HRQoL in women with fibromyalgia. These associations were generally independent of MVPA.

## Introduction

Fibromyalgia is a chronic and heterogeneous condition characterized by pain as the dominant symptom, which is frequently accompanied by fatigue, sleep disorders, or cognitive impairment [1]. Fibromyalgia patients, who tend to be highly sedentary, usually reduce their physical activity (PA) levels in order to avoid an aggravation of their symptomatology [2,3]. However, adopting this behavior might trigger a worsening of their condition [4-8]. Importantly, the risks of a sedentary lifestyle are present irrespective of the PA performed [9,10]. Considering that few patients with fibromyalgia fulfil the recommended level of moderate-to-vigorous PA (MVPA) [11], these patients are at an increased health risk not only for being highly sedentary but also for being inactive [12,13]. In the management of fibromyalgia, a graduated approach first focused on nonpharmacological modalities, and the improvement of health-related quality of life (HRQoL) is currently recommended [14]. Therefore, greater insights on how modifiable factors, such as daily sedentary time (ST) and PA, are related to HRQoL among these patients are warranted.

Emerging evidence in the general population has demonstrated that not only the total amount of ST but also the pattern of accumulation of sedentary behaviors is relevant to health [15-17]. Prolonged, unbroken periods (ie, bouts) of ST might be particularly harmful [15,16] due to its relationship with detrimental effects on the metabolism [15-17]. In fibromyalgia, Ellingson et al demonstrated that both total ST, but especially sustained ST, can negatively influence pain modulation processes [18]. In addition, the frequency of sedentary bouts seems to be linked to health outcomes, with frequent interruptions in prolonged ST (ie, breaks) being beneficially related to markers of metabolic risk [19]. Although current PA recommendations emphasize the importance of reducing total ST [20], there is no information on how sedentary behavior patterns (ie, bout duration and frequency) should be modified to maximize health benefits. Sedentary patterns have been typically collected by accelerometers in the research field [17]. In contrast, mobile health (ie, mHealth) tools are more user-friendly devices that are widely used by consumers to track daily activity. These wearable devices, however, do not usually offer sedentary behavior information to users, although the inclusion of accelerometer sensors in wearable devices [21] would make it possible. Therefore, the analysis of the impact of different patterns of sustained ST on HRQoL in fibromyalgia could help in (1) the development of recommendations to reduce overall ST and in the interruption of potentially harmful bout lengths and (2) the implementation of future mHealth tools that deliver actual sedentary pattern information and potentially encourage this population to break prolonged ST.

Therefore, we aimed to examine (1) the association of the patterns of ST (ie, ST accumulated in bouts and frequency of sedentary bouts) in different bout lengths (≥10 min, ≥20 min, ≥30 min, and ≥60 min) with the HRQoL in women with fibromyalgia, (2) the combined association of total ST and sedentary bout duration with HRQoL, and (3) whether these associations are independent of MVPA.

## Methods

### Recruitment

**Figure 1:**
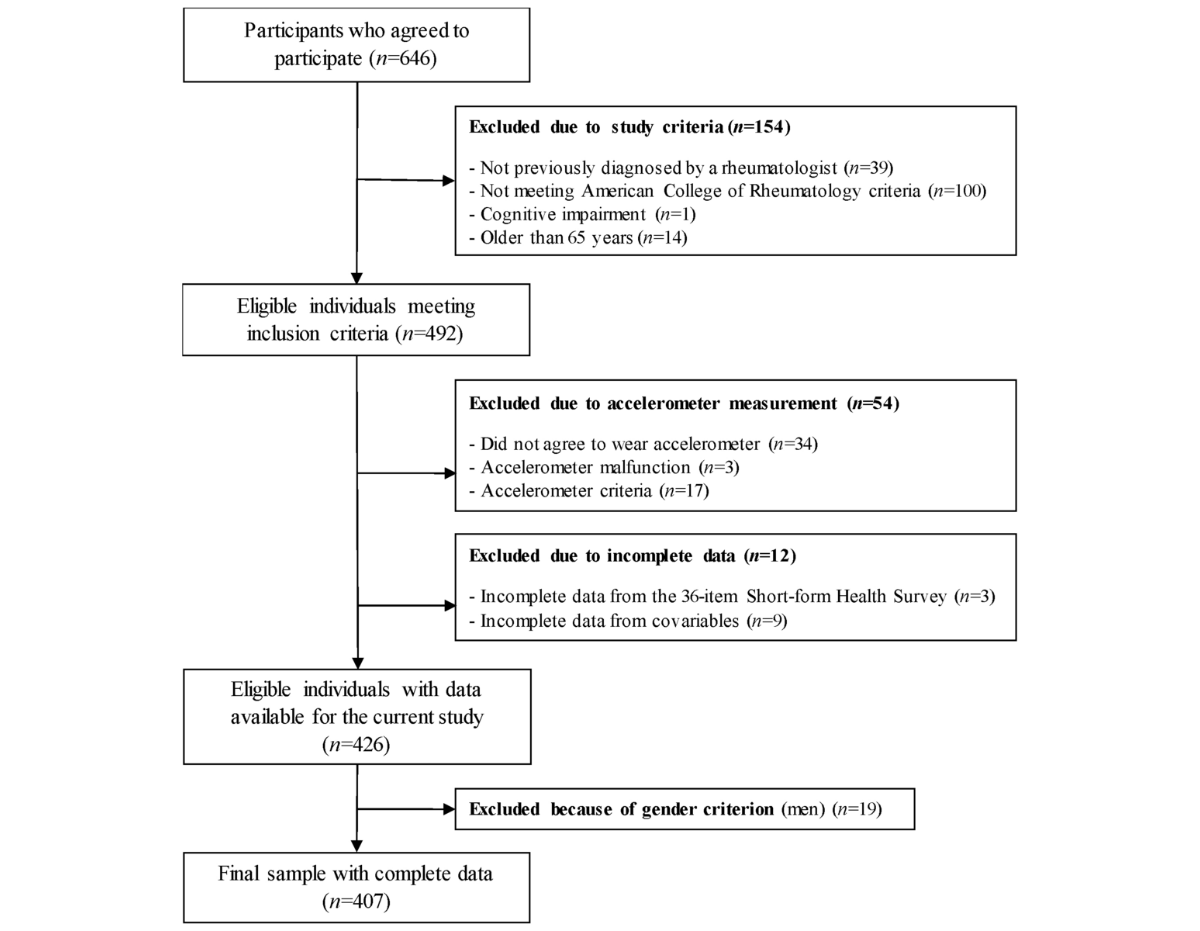
Flow diagram of inclusion of women with fibromyalgia from the al-Ándalus project included in this study (N=407).

A representative sample of fibromyalgia patients from the south of Spain—Andalusia—was recruited for the al-Ándalus project via fibromyalgia associations, internet advertisement, flyers, and email. Written informed consent from all participants (N=646) was obtained. Inclusion criteria for this study require that participants (1) be previously diagnosed by a rheumatologist and meet the 1990 American College of Rheumatology fibromyalgia criteria [1], (2) do not have either acute or terminal illness or severe cognitive impairment, and (3) are 65 years of age or younger. The flowchart of participants included in this study is shown in Figure 1. The Ethics Committee of the Hospital Virgen de las Nieves, Granada, Spain, reviewed and approved the study.

### Measurements

#### Sedentary Time and Physical Activity

Patients wore the GT3X+ triaxial accelerometer (ActiGraph) on the hip for 9 days, 24 hours per day, except during water-based activities. Activity counts were measured at a rate of 30 Hz and stored at an epoch length of 1 minute [22]. Accelerometer-wearing time was obtained by subtracting the sleeping time and nonwear periods from each day. Sleeping time was obtained from a sleep diary, in which patients reported the time they went to bed and the time they woke up. Nonwear periods were obtained by applying Choi’s algorithm [23]. Bouts of 90 continuous minutes of 0 counts were considered nonwear periods. To eliminate reactivity from the awareness of being monitored, we excluded PA data from the first day. The last day, when the device was returned, was excluded from the analysis as well. A total of 7 continuous days of recording, with a minimum of 10 valid hours per day, was the minimum criteria for being included in the study analysis.

ST and MVPA were calculated based upon recommended PA vector magnitude cut points [22,24]: 0-199 and ≥2690 counts per minute (cpm), respectively. A sedentary bout was defined as the number of consecutive minutes during which the accelerometer registered less than 200 cpm. Four sedentary bout-length categories were used in this study: ≥10 min, ≥20 min, ≥30 min, and ≥60 min. For each sedentary bout length, we obtained the following variables related to patterns of ST: (1) percentage of total ST accumulated in bouts (total time accumulated in bouts/total ST × 100) and (2) frequency of bouts (number of bouts/sedentary hours).

Data download, reduction, cleaning, and analyses were performed using ActiGraph’s desktop software: ActiLife, version 6.11.7.

#### Health-Related Quality of Life

The HRQoL was assessed using the 36-item Short-Form Health Survey (SF-36) [25]. The SF-36 is composed of 36 items that assess eight dimensions of health (ie, physical functioning, physical role, bodily pain, general health, social functioning, emotional role, mental health, and vitality) and two component summary scores (ie, physical component summary [PCS] and mental component summary [MCS]). The score in each of the eight dimensions is standardized and ranges from 0 (*worst health status*) to 100 (*best health status*).

#### Sociodemographic and Clinical Data

We collected sociodemographic and clinical data using a self-reported questionnaire that included age, marital status (married/not married), education level (university/nonuniversity), and occupational status (working/not working). Patients also reported the consumption of antidepressants and analgesics (yes/no) during the previous 2 weeks.

#### Anthropometry and Body Composition

Weight (kg) and total body fat (%) were measured using bioelectrical impedance with the InBody R20 (Biospace) body composition analyzer. Patients were asked neither to have a shower, practice intense PA, nor ingest large amounts of fluid and/or food in the 2 hours before the measurement. Patients removed their clothing and any metal objects from their bodies during the assessment.

#### Impact of the Disease

The Revised Fibromyalgia Impact Questionnaire (FIQR) [26] assesses overall fibromyalgia severity through a wide range of symptoms, comorbidities, and complaints related to this chronic condition. It is a self-administered questionnaire with 21 individual questions, with a rating scale of 0-10. The adjusted FIQR total score ranges from 0 to 100, with a higher score indicating greater impact of the syndrome on a person’s life.

### Statistical Analysis

Descriptive continuous data are shown as mean (SD), whereas categorical data are presented as n (%). To test the association between patterns of ST and HRQoL, we used linear regression analysis. The eight dimensions and the two summary components of the SF-36 were introduced as dependent variables in the models in separate regression models. Patterns of ST (ie, percentage of ST accumulated in bouts and frequency of bouts in all bout lengths) were introduced individually as predictor variables. Two types of models were built: (1) model 1 was controlled for age, total body fat percentage, occupational status, medication for pain and depression, and accelerometer wear time and (2) model 2 included model 1 plus MVPA.

The combined association of total ST and prolonged sedentary bout duration with HRQoL was studied through analyses of covariance. The subject pool was divided into four groups according to the median value of total ST (3216 min/week) and the median value of sedentary bout durations of 30 continuous minutes or more (47.7 min). A minimum duration of 30 continuous minutes was used to define prolonged ST following the criteria of previous studies [27]. The four groups created were (1) low total ST (≤ the median value) + low sedentary bout duration (≤ the median value), (2) low total ST + high sedentary bout duration (> the median value), (3) high total ST (> the median value) + low sedentary bout duration, and (4) high total ST + high sedentary bout duration. The analyses were adjusted for age, total body fat percentage, occupational status, medication for pain and depression, and accelerometer-wear time. Additional analyses including MVPA as covariate were performed.

For analyses, we used IBM SPSS Statistics for Windows, version 20.0 (IBM Corp). The statistical significance was set at *P*<.05.

### Data Exclusion

The final sample size included in the analyses comprised 407 women with fibromyalgia. The flow diagram of women with fibromyalgia included in this study is shown in Figure 1.

## Results

Table 1 provides an overview of the patients’ sociodemographic and clinical characteristics. Table 2 includes the information related to PA and ST pattern characteristics (ie, percentage of total ST and frequency of bouts) in different bout lengths.

The association of the percentage of ST accumulated in bouts of different lengths with the SF-36 domains are shown in Table 3. Greater percentages of ST spent in all bout lengths were associated with worse physical function, bodily pain, vitality, and social function domains and the PCS (beta from -.20 to -.10, all *P*<.05). In addition, a higher percentage of ST spent in bouts of 60 minutes or more was related to a worsened physical role (beta=-.10, *P*=.04). Overall, these associations were independent of MVPA (all *P*<.05), except for the bodily pain (for bouts ≥10, ≥20, or ≥30 min) and physical role domains.

**Table 1:** Sociodemographic and clinical characteristics of the study participants (N=407).

Variables		Mean (SD) or n (%)
Age (years), mean (SD)		51.4 (7.6)
Algometer score (18-144), mean (SD)		43.2 (13.4)
Body mass index (kg/m^2^), mean (SD)		28.4 (5.4)
Total body fat (%), mean (SD)		40.1 (7.6)
FIQR^a^ score (0-100), mean (SD)		64.4 (16.7)
**Health-related quality of life, SF-36** ^b^ **score (0-100), mean (SD)**		
	Physical function	39.2 (18.9)
	Physical role	33.2 (21.2)
	Bodily pain	21.2 (14.7)
	General health	28.5 (15.3)
	Vitality	22.3 (17.7)
	Social functioning	43.7 (24.7)
	Emotional role	56.9 (27.9)
	Mental health	46.2 (19.7)
	Physical component	29.5 (6.9)
	Mental component	36.0 (11.6)
**Marital status, n (%)**		
	Married	311 (76.4)
	Not married	96 (23.6)
**Education level, n (%)**		
	Nonuniversity	349 (85.7)
	University	58 (14.3)
**Current occupational status, n (%)**		
	Working	107 (26.3)
	Not working	300 (73.7)
**Drug consumption, n (%)**		
	Analgesics	367 (90.2)
	Antidepressants	232 (57.0)

^a^FIQR: Revised Fibromyalgia Impact Questionnaire.

^b^SF-36: 36-item Short-Form Health Survey.

**Table 2:** Sedentary patterns and physical activity (PA) variables of the study participants (N=407).

Sedentary behavior and PA			Mean (SD)
Accelerometer-wear time			923.0 (78.9)
**Sedentary time (ST)**			
	Minutes per day		460.1 (104.1)
	Percentage of wear time		49.9 (10.6)
**Light PA**			
	Minutes per day		418.6 (91.8)
	Percentage of wear time		45.3 (9.1)
**Moderate PA**			
	Minutes per day		43.9 (29.5)
	Percentage of wear time		4.8 (3.2)
**Vigorous PA**			
	Minutes per day		0.4 (2.0)
	Percentage of wear time		0.1 (0.2)
**Moderate-to-vigorous PA**			
	Minutes per day		44.3 (30.1)
	Percentage of wear time		4.8 (3.2)
**Patterns of ST of different bout lengths**			
	≥**10-minute bout**		
		Percentage of total ST accumulated (%)	59.2 (11.2)
		Frequency of bouts (number of bouts/week)	83.7 (25.6)
	≥**20-minute bout**		
		Percentage of total ST accumulated (%)	38.5 (12.8)
		Frequency of bouts (number of bouts/week)	34.3 (14.6)
	≥**30-minute bout**		
		Percentage of total ST accumulated (%)	26.7 (12.3)
		Frequency of bouts (number of bouts/week)	17.9 (9.6)
	≥**60-minute bout**		
		Percentage of total ST accumulated (%)	10.3 (8.9)
		Frequency of bouts (number of bouts/week)	4.3 (3.7)

**Table 3:** Association of the percentage of sedentary time (ST) accumulated in bouts of different lengths with 36-item Short-Form Health Survey (SF-36) dimensions (N=407).

Dimensions and models		Percentage of ST accumulated in bouts of different lengths															
		≥10-minute bout				≥20-minute bout				≥30-minute bout				≥60-minute bout			
		B^a^	SE	Beta^b^	*P*	B	SE	Beta	*P*	B	SE	Beta	*P*	B	SE	Beta	*P*
**Physical function**																	
	Model 1^c^	-0.267	0.086	-.159	.002	-0.253	0.074	-.171	.001	-0.271	0.077	-.176	<.001	-0.428	0.104	-.201	<.001
	Model 2^d^	-0.226	0.089	-.134	.01	-0.221	0.077	-.149	.004	-0.239	0.079	-.156	.002	-0.396	0.105	-.186	<.001
**Physical role**																	
	Model 1	-0.126	0.094	-.067	.18	-0.137	0.081	-.082	.09	-0.159	0.084	-.092	.06	-0.239	0.114	-.100	.04
	Model 2	-0.063	0.097	-.033	.52	-0.089	0.083	-.054	.29	-0.115	0.086	-.067	.18	-0.195	0.115	-.082	.09
**Bodily pain**																	
	Model 1	-0.130	0.065	-.099	.045	-0.108	0.056	-.094	.05	-0.114	0.058	-.096	.048	-0.190	0.078	-.115	.02
	Model 2	-0.106	0.067	-.081	.12	-0.089	0.058	-.077	.13	-0.096	0.059	-.080	.11	-0.171	0.079	-.104	.03
**General health**																	
	Model 1	-0.034	0.069	-.025	.62	-0.048	0.060	-.040	.42	-0.058	0.062	-.047	.35	-0.076	0.084	-.044	.37
	Model 2	-0.029	0.072	-.021	.69	-0.046	0.062	-.038	.46	-0.056	0.064	-.045	.38	-0.073	0.085	-.042	.39
**Vitality**																	
	Model 1	-0.252	0.080	-.160	.002	-0.204	0.069	-.148	.003	-0.204	0.072	-.142	.004	-0.278	0.097	-.140	.004
	Model 2	-0.209	0.083	-.133	.01	-0.168	0.071	-.122	.02	-0.169	0.073	-.117	.02	-0.242	0.098	-.122	.01
**Social functioning**																	
	Model 1	-0.399	0.106	-.181	<.001	-0.351	0.091	-.182	<.001	-0.361	0.095	-.180	<.001	-0.500	0.129	-.181	<.001
	Model 2	-0.285	0.108	-.130	.01	-0.261	0.093	-.135	.01	-0.275	0.095	-.137	.004	-0.412	0.128	-.149	.001
**Emotional role**																	
	Model 1	0.040	0.121	.016	.74	0.012	0.105	.005	.91	-0.016	0.109	-.007	.88	-0.034	0.148	-.011	.82
	Model 2	0.077	0.126	.031	.54	0.039	0.109	.018	.72	0.008	0.112	.004	.94	-0.010	0.150	-.003	.95
**Mental health**																	
	Model 1	0.028	0.086	.016	.74	-0.015	0.075	-.010	.84	-0.029	0.077	-.018	.71	-0.104	0.105	-.047	.32
	Model 2	0.059	0.090	.034	.51	0.006	0.077	.004	.94	-0.010	0.079	-.006	.90	-0.087	0.106	-.039	.41
**Physical component**																	
	Model 1	-0.096	0.032	-.156	.003	-0.085	0.027	-.158	.002	-0.089	0.028	-.159	.002	-0.130	0.038	-.168	.001
	Model 2	-0.083	0.033	-.135	.01	-0.075	0.028	-.139	.01	-0.079	0.029	-.141	.01	-0.119	0.039	-.154	.002
**Mental component**																	
	Model 1	-0.023	0.050	-.022	.64	-0.031	0.043	-.034	.47	-0.039	0.045	-.041	.39	-0.065	0.061	-.050	.28
	Model 2	0.003	0.052	.003	.95	-0.011	0.044	-.012	.80	-0.020	0.046	-.021	.66	-0.047	0.061	-.036	.44

^a^B: nonstandardized regression coefficient. Linear regression models were built using *Enter* method, with SF-36 domains as dependent variables and percentage of ST in different bout lengths as independent variables.

^b^Beta: standardized regression coefficient.

^c^Model 1: adjusted for age, fat percentage, occupational status, medication for pain, medication for depression, and accelerometer wear time.

^d^Model 2: analysis controlled for model 1 + moderate-to-vigorous PA.

Table 4 shows the association of the frequency of bouts of ST of different lengths with the SF-36 domains. A higher frequency of sedentary bouts 20 minutes or longer was associated with worsened vitality and social function (beta=-.12 and -.13, respectively, all *P*<.05). A higher frequency of sedentary bouts 30 minutes or longer was associated with worsened physical function, vitality, social function, and PCS scores (beta from -.15 to -.12, all *P*<.05). A higher frequency of sedentary bouts 60 minutes or longer was associated with worsened physical function, physical role, bodily pain, vitality, social function, and PCS scores (beta from -.19 to -.10, all *P*<.05). These associations were independent of MVPA, except for the association with physical role, vitality, and social function in bouts 20 minutes or longer.

**Figure 2:**
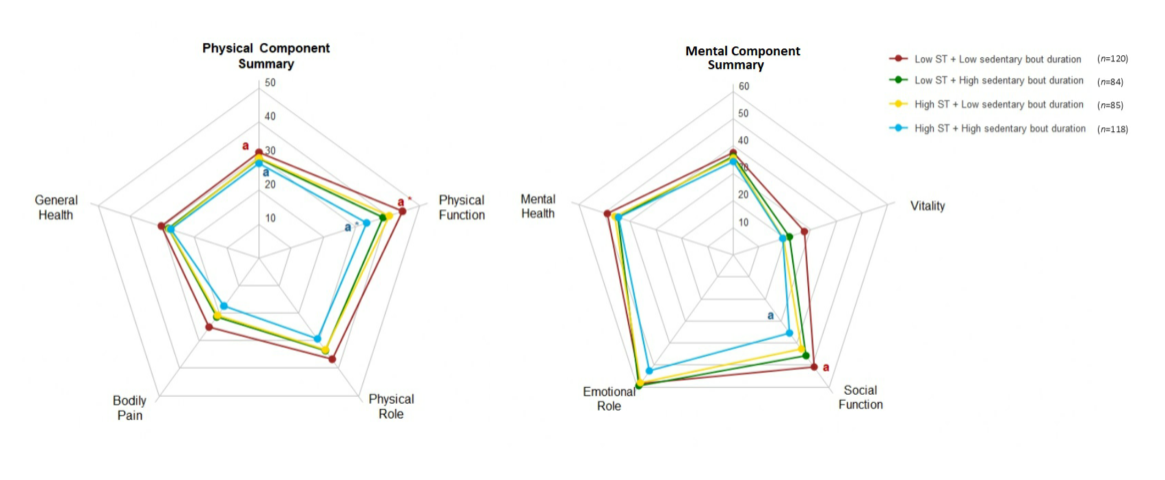
Combined association of total sedentary time (ST) and prolonged sedentary bouts of at least 30 minutes with health-related quality of life. Estimated means represent values after adjustment for age, total body fat percentage, occupational status, medication for pain, medication for depression, and accelerometer-wear time. Common superscripts indicate significant (*P*≤.05) differences between groups with the same letter when adjusting for age, body fat percentage, occupational status, medication for pain, medication for depression, and accelerometer-wear time. Asterisks represent significant differences for additional adjustment for moderate-to-vigorous physical activity (*P*=.045).

Figure 2 shows the combined association of total ST and sedentary bout duration with the SF-36 domains, the PCS, and the MCS. Participants with low total ST and low sedentary bout duration presented better physical function (mean difference 8.73 units, 95% CI 2.31-15.15), social function (mean difference 10.51 units, 95% CI 2.59-18.44), and PCS (mean difference 2.71 units, 95% CI 0.36-5.06) compared to participants with high total ST and high sedentary bout duration (all *P*<.02). Additional analyses showed that the differences in the physical function (*P*=.045) were independent of MVPA.

**Table 4:** Association of the frequency of bouts of sedentary time (ST) of different lengths with 36-item Short-Form Health Survey (SF-36) dimensions (N=407).

Dimensions and models		Frequency of bouts (number of bouts/sedentary hours) of different bout lengths															
		≥10-minute bout				≥20-minute bout				≥30-minute bout				≥60-minute bout			
		B^a^	SE	Beta^b^	*P*	B	SE	Beta	*P*	B	SE	Beta	*P*	B	SE	Beta	*P*
**Physical function**																	
	Model 1^c^	2.386	4.237	.028	.57	-10.754	5.882	-.092	.07	-18.793	7.663	-.123	.02	-61.611	16.061	-.188	<.001
	Model 2^d^	3.728	4.240	.044	.38	-7.970	6.002	-.068	.19	-15.416	7.820	-.101	.049	-56.713	16.208	-.173	.001
**Physical role**																	
	Model 1	3.602	4.575	.038	.43	-4.921	6.375	-.038	.44	-12.379	8.315	-.072	.14	-35.905	17.572	-.098	.04
	Model 2	5.161	4.572	.055	.26	-1.341	6.490	-.010	.84	-8.033	8.469	-.047	.34	-29.379	17.693	-.080	.10
**Bodily pain**																	
	Model 1	-1.811	3.163	-.028	.57	-6.140	4.399	-.068	.16	-9.957	5.741	-.084	.08	-31.088	12.109	-.122	.01
	Model 2	-1.128	3.179	-.017	.72	-4.636	4.500	-.051	.30	-8.101	5.873	-.068	.17	-28.355	12.245	-.111	.02
**General health**																	
	Model 1	1.978	3.370	.029	.56	-2.700	4.696	-.029	.57	-6.395	6.132	-.052	.30	-14.374	12.987	-.054	.27
	Model 2	2.167	3.399	.032	.52	-2.433	4.818	-.026	.61	-6.189	6.289	-.050	.33	-13.956	13.166	-.053	.29
**Vitality**																	
	Model 1	-4.226	3.928	-.053	.28	-13.300	5.441	-.122	.02	-19.025	7.101	-.133	.01	-44.261	15.015	-.145	.003
	Model 2	-2.976	3.931	-.038	.45	-10.617	5.549	-.097	.06	-15.624	7.240	-.109	.03	-38.871	15.125	-.127	.01
**Social functioning**																	
	Model 1	-2.477	5.242	-.022	.64	-19.997	7.237	-.132	.01	-30.153	9.429	-.151	.001	-73.977	19.887	-.173	<.001
	Model 2	0.493	5.150	.004	.92	-13.339	7.267	-.088	.07	-21.717	9.472	-.109	.02	-60.962	19.730	-.143	.002
**Emotional role**																	
	Model 1	4.353	5.913	.035	.46	1.973	8.246	.011	.81	-2.809	10.777	-.012	.79	-4.716	22.830	-.010	.84
	Model 2	5.130	5.958	.041	.39	3.877	8.449	.023	.65	-0.610	11.041	-.003	.96	-1.328	23.120	-.003	.95
**Mental health**																	
	Model 1	5.702	4.196	.065	.18	0.485	5.861	.004	.93	-2.013	7.659	-.013	.79	-19.328	16.197	-.057	.23
	Model 2	6.372	4.225	.072	.13	1.989	6.003	.016	.74	-0.195	7.844	-.001	.98	-16.941	16.404	-.050	.30
**Physical component**																	
	Model 1	-0.238	1.557	-.008	.88	-4.104	2.160	-.097	.06	-6.827	2.816	-.122	.02	-19.387	5.930	-.162	.001
	Model 2	0.186	1.562	.006	.91	-3.222	2.208	-.076	.15	-5.754	2.877	-.103	.046	-17.763	5.989	-.149	.003
**Mental component**																	
	Model 1	1.309	2.431	.025	.59	-1.825	3.387	-.025	.59	-4.017	4.424	-.043	.36	-11.036	9.365	-.055	.24
	Model 2	1.910	2.440	.037	.43	-0.438	3.461	-.006	.90	-2.300	4.520	-.024	.61	-8.442	9.459	-.042	.37

^a^B: nonstandardized regression coefficient. Linear regression models built using *Enter* method, with SF-36 domains as dependent variables and percentage of ST in different bout lengths as independent variables.

^b^Beta: standardized regression coefficient.

^c^Model 1: adjusted for age, fat percentage, occupational status, medication for pain, medication for depression, accelerometer wear time, and ST.

^d^Model 2: analysis controlled for model 1 + moderate-to-vigorous PA.

## Discussion

### Principal Findings

The main findings of this study suggest that higher percentages of ST spent in different bout lengths were associated with worsened HRQoL, including physical function, bodily pain, vitality, and social function domains, as well as the PCS. Also, higher frequencies of sedentary bouts were associated with worsened HRQoL, including physical function, bodily pain, vitality, and social function domains, as well as the PCS, especially in longer bout durations. Patients characterized by high total ST and high sedentary bout duration presented worsened physical function, social function, and PCS scores. These associations were generally independent of the MVPA performed for long bout lengths. These findings entail a first step toward the understanding of free-living sedentary behavior and its association with HRQoL in fibromyalgia. This supports the implementation of mHealth devices, which allow self-monitoring and immediate feedback of daily living behaviors to patients. Future studies might determine whether this approach is successful by reducing prolonged ST in this population.

### Limitations

This study has several limitations that should be underlined. Because our results were derived from a cross-sectional design, the associations cannot be explained via a causal pathway. In addition, due to the large quantity of factors related to HRQoL, it is difficult to ascertain the true nature of the association found between variables. Because only women took part in this study, future studies should investigate whether these associations might extend to men as well. Among its strengths, this study includes a relatively large sample size of women with fibromyalgia representative from the south of Spain (ie, Andalusia). According to a recent study, measurement of the actual dose of exercise and daily mobility are essential to establish relationships of these behaviors with health [21]. In this sense, ST and PA were objectively assessed in this study through a wearable tool that enabled researchers to monitor the type, quantity, and quality of everyday activities of patients via accelerometry, which is considered a more reliable technique than questionnaires in the study of fibromyalgia [28]. Future intervention studies with mHealth devices that incorporate in situ information are warranted in this population to ascertain whether fibromyalgia patients change their sedentary behaviors.

### Comparison With Prior Work

To date, most of the previous research on ST and health in fibromyalgia has been limited to the study of total ST [7,29]. In addition, few studies have objectively characterized ST through accelerometry in these patients [18,30] and only one of them [18] reported the values of sustained ST (>1 hour). Ellingson et al demonstrated that sustained ST (>60 min) was associated with worse pain modulation in fibromyalgia—assessed through magnetic resonance imaging—to a greater extent compared to total ST [18]. Congruently, this study showed negative associations between time spent in sedentary bouts (≥10 min, ≥20 min, ≥30 min, and ≥60 min) and the SF-36 body pain dimension. Therefore, we extend the connection between sustained ST and pain to patient-reported instruments. Also, the interruptions of these sedentary bouts might be relevant for pain in this population, given that frequency of sedentary bouts (≥60 min) was negatively associated with bodily pain scores. Following the findings by Ellingson et al, increased pain in sustained ST could be due to the impaired activity in the prefrontal cortices and sensory regions (ie, pre- and postcentral gyri) of these patients [18]. Because the bodily pain domain of the SF-36 not only encompasses objective levels of pain but also the perceived limitations due to it, the contribution of other factors influencing patients’ perceptions, such as self-efficacy or pain coping strategies [31], could also take part in this relationship.

Although the influence of patterns of ST has not been explored in fibromyalgia, a direct relationship between increased total ST and fatigue has been described [29]. In agreement with our findings, one previous study in healthy women showed that prolonged ST accumulated in bouts of at least 1 hour were negatively associated with vitality scores of the SF-36 and other fatigue-related variables [32]. Despite the cross-sectional design of these findings that precludes the causal explanation, other experimental studies observed increases in fatigue levels during uninterrupted sitting in adults with overweight and obese status [33] and type 2 diabetes [34], or decreases in fatigue as a result of reducing prolonged sitting [33]. The relationship between ST and fatigue might be explained through physiological, psychological, and social factors that contribute to this multifaceted phenomenon. For instance, prolonged ST could alter the sympathetic nervous system (ie, through a lower heart rate, decreased plasma level of dihydroxyphenylalanine, and increased plasma level of dihydroxyphenylglycol) [33], promote muscle fatigue through sustained activation of low-threshold motor units in sedentary positions [35], or negatively influence sleep quality [7].

In fibromyalgia, there is also a gap in the literature regarding the influence of ST and its patterns on social limitations due to health. For other social-related aspects, Soursa et al stated that patients with fibromyalgia with the lowest PA levels and, presumably, higher levels of ST, had fewer social interactions compared to those doing more PA [36]. No evidence is available on how patterns of ST could influence social function in other populations either, yet interpersonal factors (eg, family, friends, and social networks) are well-known determinants of sedentary behaviors [37]. The passive nature of different sedentary activities (eg, watching television or sitting at the computer) that are accompanied with decreased communication [38] could also lead to poor social networking and participation [39]. Therefore, future research might ascertain whether breaking prolonged ST could positively influence this construct of health (eg, through an increased opportunity to interact with others) or whether strategies aimed at increasing social support may lead to more favorable patterns of accumulation of ST.

To our knowledge, no previous studies have linked patterns of ST to physical function in fibromyalgia. The physical function domain assesses activities of daily living (eg, bathing, dressing, walking several blocks, and lifting or carrying groceries) that typically require a combination of flexibility, strength, and cardiorespiratory fitness, which are related to HRQoL [40]. Previous evidence in adults or older adults showed a decreased physical function, assessed through physical fitness tests, in relation to more deleterious patterns of device-measured ST, such as reduced breaks in ST [41,42], increased sedentary bout duration [41], or increased total prolonged ST [42]. Sedentary periods are linked to skeletal muscle inactivity [43] and are thought to accelerate sarcopenia and loss of aerobic capacity [44], which could negatively affect physical function. Therefore, increases in physical function could be optimized by avoiding the accumulation of ST in prolonged periods and reducing the duration of these ST periods, which needs to be confirmed in future intervention studies.

We observed that, overall, the strength of the associations between the patterns of ST and HRQoL was reduced but still significant when considering MVPA. This finding is congruent with a recent meta-analysis concluding that the deleterious health effects associated with ST generally decrease in magnitude among people with higher levels of PA [13]. Our results also showed that, for certain patterns of ST in shorter bout lengths (<60 min), the associations with HRQoL were not significant anymore when considering MVPA. Therefore, performing MVPA could have a protective effect only when ST is accrued in low-duration bouts and could be especially relevant for certain domains of HRQoL, such as bodily pain, physical role, vitality, or social function. Interestingly, meeting the current guidelines of MVPA in bouts of at least 10 minutes was found to neutralize the negative association of prolonged ST with fatigue in healthy women [32]. Hence, it is possible that the patterns of accumulation of MVPA could also influence the capacity of this behavior of counteracting the negative effects of prolonged ST.

### Conclusions

In conclusion, our findings indicate that higher ST spent in diverse bout lengths and a higher frequency of sedentary bouts, especially in longer bout durations, is associated with worsened HRQoL, more specifically with physical function, bodily pain, vitality, and social function domains, as well as the PCS. Patients that are highly sedentary and present longer sedentary bout durations have worsened physical function, social function, and PCS scores. Although these associations were generally independent of MVPA in long sedentary bout lengths, this intensity of PA could play a positive role when ST is accumulated in shorter bouts. Future intervention studies using mHealth devices that incorporate immediate feedback for users are warranted in this population to ascertain whether fibromyalgia patients change their sedentary behaviors.

## Abbreviations

cpm: counts per minute
ERDF: European Regional Development Fund
FIQR: Revised Fibromyalgia Impact Questionnaire
HRQoL: health-related quality of life
MCS: mental component summary
MVPA: moderate-to-vigorous physical activity
PA: physical activity
PAHELP: Physical Activity for Health Promotion
PCS: physical component summary
SF-36: 36-item Short-Form Health Survey
ST: sedentary time
UCEES: Unit of Excellence on Exercise and Health
